# C-reactive protein may be a prognostic factor in hepatocellular carcinoma with malignant portal vein invasion

**DOI:** 10.1186/1477-7819-11-92

**Published:** 2013-04-23

**Authors:** Jong Man Kim, Choon Hyuck David Kwon, Jae-Won Joh, Justin Sangwook Ko, Jae Berm Park, Joon Hyeok Lee, Sung Joo Kim, Seung Woon Paik, Cheol-Keun Park

**Affiliations:** 1 Department of Surgery, Samsung Medical Center, Sungkyunkwan University School of Medicine, #50 Ilwon-Dong Gangnam-Gu, Seoul, 135-710, Korea; 2 Department of Anesthesiology and Pain Medicine, Samsung Medical Center, Sungkyunkwan University School of Medicine, Seoul, 135-710, Korea; 3 Division of Gastroenterology, Department of Medicine, Samsung Medical Center, Sungkyunkwan University School of Medicine, Seoul, 135-710, Korea; 4 Department of Pathology, Samsung Medical Center, Sungkyunkwan University School of Medicine, Seoul, 135-710, Korea

**Keywords:** C-reactive protein, Hepatectomy, Hepatocellular carcinoma, Malignant portal vein invasion, Tumor recurrence

## Abstract

**Background:**

Hepatocellular carcinoma (HCC) has a high predilection for portal vein invasion, and the prognosis of HCC with malignant portal vein invasion is extremely poor. The objective of this study was to investigate the outcomes and the prognostic factor of recurrence in HCC patients with malignant portal vein invasion.

**Methods:**

We retrospectively reviewed the clinicopathologic data and outcomes of 83 HCC patients with malignant portal vein invasion and 1,056 patients without portal vein invasion who underwent liver resection.

**Results:**

Increased serum alkaline phosphatase (ALP) levels, increased maximum tumor size, and intrahepatic metastasis were predisposing factors for malignant portal vein invasion by multivariate analysis. The median disease-free survival and overall survival of HCC patients with malignant portal vein invasion was 4.5 months and 25 months, respectively. The 1-year, 2-year, and 3-year disease-free survival rates were 30.6%, 26.1%, and 21.2%, respectively, and the overall survival rates for HCC patients with malignant portal vein invasion were 68.6%, 54.2%, and 41.6%, respectively. The initial detection site was the lung in HCC patients with portal vein invasion and the liver in HCC patients without portal vein invasion. C-reactive protein (CRP) was a significant independent predictor of tumor recurrence in HCC with malignant portal vein invasion after surgery.

**Conclusions:**

Increased ALP levels, increased maximum tumor size, and intrahepatic metastasis were independent predictors of malignant portal vein invasion in HCC. CRP level was closely associated with the predisposing factor of tumor recurrence in HCC patients with malignant portal vein invasion after a surgical resection, and lung metastasis was common.

## Background

Hepatocellular carcinoma (HCC) is a leading cause of cancer death, and its incidence is particularly high in Asian countries, including Korea. Chronic hepatitis B is endemic in Korea, representing the most important risk factor and constituting approximately 70% of all HCC cases [1].

The prognosis of HCC is very poor as a result of intrahepatic metastasis and recurrence, which are closely associated with portal vein invasion [2,3]. HCC has a tendency to invade the portal vein causing tumor thrombosis, which has been shown to be an adverse prognostic factor for HCC [4]. The prognosis of HCC with portal vein tumor invasion is extremely poor, with a median survival of 2.7 to 4 months without intervention [5]. However, the characteristics of HCC with malignant portal vein invasion are not well understood.

Although liver transplantation for HCC alters the natural history of this disease, liver resection remains one of the main treatment modalities that can provide a good outcome [6].

In this study, we retrospectively compared patients with portal vein invasion to patients without portal vein invasion and analyzed the predisposing factors of tumor recurrence in HCC patients with malignant portal vein invasion who underwent liver resection.

## Methods

### Patients

From January 2006 to June 2010, 1,139 patients who were newly diagnosed with HCC underwent liver resection at Samsung Medical Center. The diagnoses of HCC with malignant portal vein invasion were confirmed by a histologic examination after surgery. We excluded patients who were younger (age <18 years), had pathologically proven mixed hepatocellular carcinoma and cholangiocarcinoma, or who were lost to follow-up after liver resection. The demographic, preoperative laboratory, and pathologic data of all patients were collected from the electronic medical record (EMR) and retrospectively reviewed. The Child-Pugh classification system was used to evaluate liver function.

### Surgery and pathology

Selection criteria for the liver resection procedure depended on tumor location and extent, liver function, and future liver remnant volume. Child-Pugh class C, severe co-morbidity, and distant metastasis were considered contraindications for hepatectomy. A standard operative technique for the hepatectomy was used for these tumors. Depending on the part of liver to be resected, adequate mobilization was performed. Selective clamping of the portal vein and hepatic artery was performed when feasible; if not, intermittent the Pringle maneuver was performed. The parenchymal transection was performed using Cavitron Ultrasonic Surgical Aspirator (CUSA) under low central venous pressure. A major hepatectomy was defined as a resection of three or more Couinaud segments, and minor hepatectomy was defined as resection of less than three segments. R_0_ resection status was defined as the presence of microscopic tumor >1 mm from the resection margin.

Postoperative histological assessment and reporting included the maximal tumor diameter, capsular formation, capsular invasion, portal vein invasion, bile duct invasion, microvascular invasion, serosal involvement, intrahepatic metastasis, multicentric occurrence of HCC, cirrhosis, resection margin, and others. Histologic grade of HCC was assessed according to the Edmonson-Steiner grade system, and groups as well differentiated (grade I), moderate differentiated (grade II), or poorly differentiated (grades III, IV) [7].

### Surveillance after surgical resection

After surgery, the patients with HCC with pathologic portal vein invasion were followed up every 2 to 3 months in the postoperative period. A follow-up included physical examination, serum alpha-fetoprotein (AFP), protein induced by vitamin K antagonist (PIVKA)-II, liver function test, and chest X-ray. An abdominal computed tomography (CT) was performed every 3 months or when intrahepatic recurrence was suspected. Magnetic resonance imaging (MRI) and/or positron emission tomography (PET) scan were performed as CT could not show definitively the evidence of recurrence. Detailed information on patients who were identified to have peritoneal recurrence was recorded. Patients with intrahepatic recurrence were treated with radiofrequency ablation (RFA), transarterial chemoembolization (TACE), or sorafenib according to their liver function reserve and the pattern of recurrence. The follow-up time was defined as the length of time from surgery to last follow-up (December 1, 2011) or death. None of the patients were lost to follow-up or died within 30 days after surgery, and 1,139 patients were included in the survival analysis.

### Statistical analysis

All data were analyzed using SPSS statistical software (Ver 19.0; SPSS Inc., Chicago, IL, USA). Continuous variables are presented as the median and range and were compared by Mann–Whitney *U* test. Categorical variables were compared by Fisher’s exact test. A multivariate analysis was performed to identify the risk factors of the pathological portal vein invasion using logistic regression analysis. The disease-free survival rates and overall survival rates were calculated with the Kaplan-Meier method and compared using the log-rank test. Univariate and multivariate analyses were performed to identify the risk factors of HCC recurrence in HCC with pathologic portal vein invasion using Cox regression model. A *P* value of <0.05 was considered statistically significant.

## Results

### Demographics of HCC patients with portal vein invasion

The clinicopathologic features of HCC patients with malignant portal vein invasion and HCC patients without malignant portal vein invasion are summarized in Table 1. Of the 83 HCC patients (7.3%) with malignant portal vein invasion, there were 71 men and 12 women, with a median age of 49 years (range, 20–72 years). There were 1,056 (92.7%) HCC patients without portal vein invasion, and their median age was 54 years (range, 19–92 years). The age of HCC patients with malignant portal vein invasion was lower than that of HCC patients without portal vein invasion (*P* <0.001). Most cases of HCC were caused by hepatitis B virus (HBV), and patients with malignant portal vein invasion did not receive radiation in the preoperative period. There were no differences in gender, cause, and type of locoregional therapy between the two groups. As for laboratory data before the operation, the HCC patients with malignant portal vein invasion had higher serum aspartate aminotransferase (AST), alanine aminotransferase (ALT), alkaline phosphatase (ALP), gamma-glutamyl transferase (GGT), estimated glomerular filtration rate (GFR), and C-reactive protein (CRP) levels compared with HCC patients without portal vein invasion (*P* <0.05). However, white blood cell (WBC) count, hemoglobin, platelet count, INR, albumin, total bilirubin, direct bilirubin, and creatinine were similar in both groups. The median of AFP and PIVKA-II in HCC patients with malignant portal vein invasion was 813.2 ng/mL (range, 1.8–200,000 ng/mL) and 482.0 mAU/mL (range, 12–1,200 mAU/mL), respectively, but the median in HCC patients without portal vein invasion was 26.4 ng/mL (range, 1.0–2,121,720 ng/mL) and 42.0 mAU/mL (range, 2–2,000 mAU/mL), respectively (*P* <0.001 and *P* <0.001, respectively).

**Table 1:** Characteristics of HCC patients with and without portal vein invasion

**Variables**	**HCC with PV invasion** **(*n*=83, 7.3%)**	**HCC without PV invasion** **(*n*=1056, 92.7%)**	***P*value**
*Gender*			0.252
Male	71 (85.5%)	842 (79.7%)	
Female	12 (14.5%)	214 (20.3%)	
Age (years)	49 (20–72)	54 (19–82)	<0.001
*Etiology*			0.447
HBV	63 (75.9%)	816 (77.6%)	
HCV	4 (4.8%)	54 (5.1%)	
Alcohol	4 (4.8%)	19 (1.8%)	
Non-B, non-C	10 (12.0%)	142 (13.5%)	
Others	2 (2.4%)	20 (1.9%)	
*Locoregional therapy*			0.070
None	64 (77.1%)	907 (85.9%)	
TACE	18 (21.7%)	127 (12.0%)	
RFA	1 (1.2%)	13 (1.2%)	
TACE and RFA	0 (0%)	9 (0.9%)	
White blood cells (/uL)	5,730 (2,520–10,530)	5,460 (1,270–16,530)	0.103
Hemoglobin (g/dL)	14.3 (9.8–17.0)	14.2 (8.1–16.8)	0.360
Platelet counts (/uL)	174,000 (80,000–627,000)	160,000 (19,000–708,000)	0.125
INR	1.09 (0.93–1.32)	1.08 (0.84–1.59)	0.901
Albumin (g/dL)	4.1 (2.9–5.0)	4.1 (2.1–5.3)	0.423
Total bilirubin (mg/dL)	0.7 (0.2–3.5)	0.7 (0.2–4.3)	0.310
AST (U/L)	43 (23–283)	35 (14–353)	<0.001
ALT (U/L)	44 (17–288)	35 (13–385)	0.002
ALP (U/L)	93 (38–233)	79 (32–562)	<0.001
GGT (U/L)	105 (17–1,442)	49 (8–1,322)	<0.001
Creatinine (mg/dL)	0.86 (0.44–1.27)	0.88 (0.42–5.87)	0.586
Estimated GFR (mL/min)	98.6 (72.2–149.2)	89.4 (5.7–189.0)	0.003
CRP (mg/dL)	0.27 (0.04–28.62)	0.13 (0.01–22.24)	0.048
AFP (ng/mL)	813.2 (1.8–200,000)	26.4 (1.0–2,121,720)	<0.001
PIVKA-II (mAU/mL)	482.0 (12–1,200)	42.0 (2–2,000)	<0.001
*Operation*			<0.001
Anatomical	61 (73.5%)	540 (51.1%)	
Non-anatomical	22 (26.5%)	516 (48.9%)	
*Hepatectomy*			<0.001
Major	50 (60.2%)	274 (25.9%)	
Minor	33 (39.8%)	782 (74.1%)	
*Resection*			0.035
R _0_	77 (93.9%)	1029 (98.0%)	
R _1_	5 (6.1%)	21 (2.0%)	
Margin (mm)	10 (1–53)	10 (1–80)	0.331
Maximum tumor size (mm)	6.0 (1.4–17.5)	3.3 (0.2–21.0)	<0.001
*Grade*			0.001
1 and 2	67 (80.7%)	976 (92.4%)	
3 and 4	16 (19.3%)	80 (7.6%)	
Cirrhosis	40 (48.2%)	468 (44.3%)	0.494
*Capsule formation*			<0.001
None	33 (39.8%)	69 (6.6%)	
Complete	35 (42.2%)	875 (83.2%)	
Partial	15 (18.1%)	108 (10.3%)	
Bile duct invasion	7 (8.4%)	24 (2.3%)	0.005
Serosa involvement	2 (2.4%)	12 (1.1%)	0.272
Intrahepatic metastasis	41 (49.4%)	112 (10.6%)	<0.001
Multicentric occurrence	1 (1.2%)	57 (5.4%)	0.118
Hospitalization	9 (5–53)	9 (3–102)	0.932
Duration of Follow-up (months)	18 (5–68)	32 (3–82)	<0.001

AFP, Alpha-fetoprotein; ALP, Alkaline phosphatase; ALT, Alanine aminotransferase; AST, Aspartate aminotransferase; CRP, C-reactive protein; GFR, Estimated glomerular filtration rate; GGT, Gamma-glutamyl transferase; HBV, Hepatitis B virus; HCV, Hepatitis C virus; INR, International normalized ratio; PIVKA-II, Protein induced by vitamin K antagonist-II; PV, Portal vein; RFA, Radiofrequency ablation; TACE, Transarterial chemoembolization.

### The surgery and pathologic results in HCC patients with malignant portal vein invasion

The proportion of anatomical resection and major hepatectomy in HCC patients with malignant portal vein invasions was higher than that in HCC patients without portal vein invasion (*P* <0.001 and *P* <0.001, respectively). Postoperative mortality was not occurred in the patients with malignant portal vein invasion. Pathologically proven malignant portal vein invasion consisted of segmental portal vein invasion (*n*=57) and left or right main portal vein invasion (*n*=26). Left or right main portal vein invasion was detected in the preoperative radiology, but segmental portal vein invasion was not diagnosed in the preoperative radiology. The median tumor size was 6 cm (range, 1.4–17.5 cm) in HCC with malignant portal vein invasion and 3.3 cm (range, 0.2–21.0 cm) in HCC without portal vein invasion (*P*<0.001). The proportion of R_1_ resection in HCC patients with malignant portal vein invasion was 6.1%, which was higher than that in HCC patients without portal vein invasion (*P*=0.035). None of the studied patients had distant metastasis and there was no patient with R_2_ resection in this study. There were 16 cases (19.3%) of grade 3 or 4 HCC with malignant portal vein invasion and 80 (7.6%) cases of HCC without portal vein invasion. The grade in HCC with malignant portal vein invasion was poorer than that in HCC without portal vein invasion (*P*=0.001). Bile duct invasion, intrahepatic metastasis, and the absence of capsular formation were more prevalent in HCC with portal vein invasion than in HCC without portal vein invasion (*P* <0.05). However, no significant difference was found in serosa involvement and multicentric occurrence between the two groups. The median hospitalization in both groups was 9 days and the duration of follow-up in HCC patients with malignant portal vein invasion was 18 months (range, 5-68 months), and that in HCC with portal vein invasion was 32 months (range, 3-82 months). The duration of follow-up in HCC patients with malignant portal vein invasion was shorter than that in HCC patients without portal vein invasion because HCC patients died due to tumor recurrence in the early period after surgery.

### Risk factors for malignant portal vein invasion in HCC patients

To identify risk factors for malignant portal vein invasion in HCC, we performed a multivariate analysis on all variables that were significantly associated with malignant portal vein invasion on univariate analysis. Increased serum ALP levels, increased maximum tumor size, and intrahepatic metastasis were the predisposing factors of malignant portal vein invasion by multivariate analysis (Table 2).

**Table 2:** Risk factors for portal vein invasion in HCC by multivariate analysis

**Variables**	**Hazard ratio**	**95% confidence interval**	***P*value**
ALP	1.017	1.005–1.030	0.007
Maximum tumor size	1.025	1.010–1.040	0.001
Intrahepatic metastasis	6.064	1.553–23.673	0.009

ALP, Alkaline phosphatase.

### Survival rate after surgery

**Figure 1:**
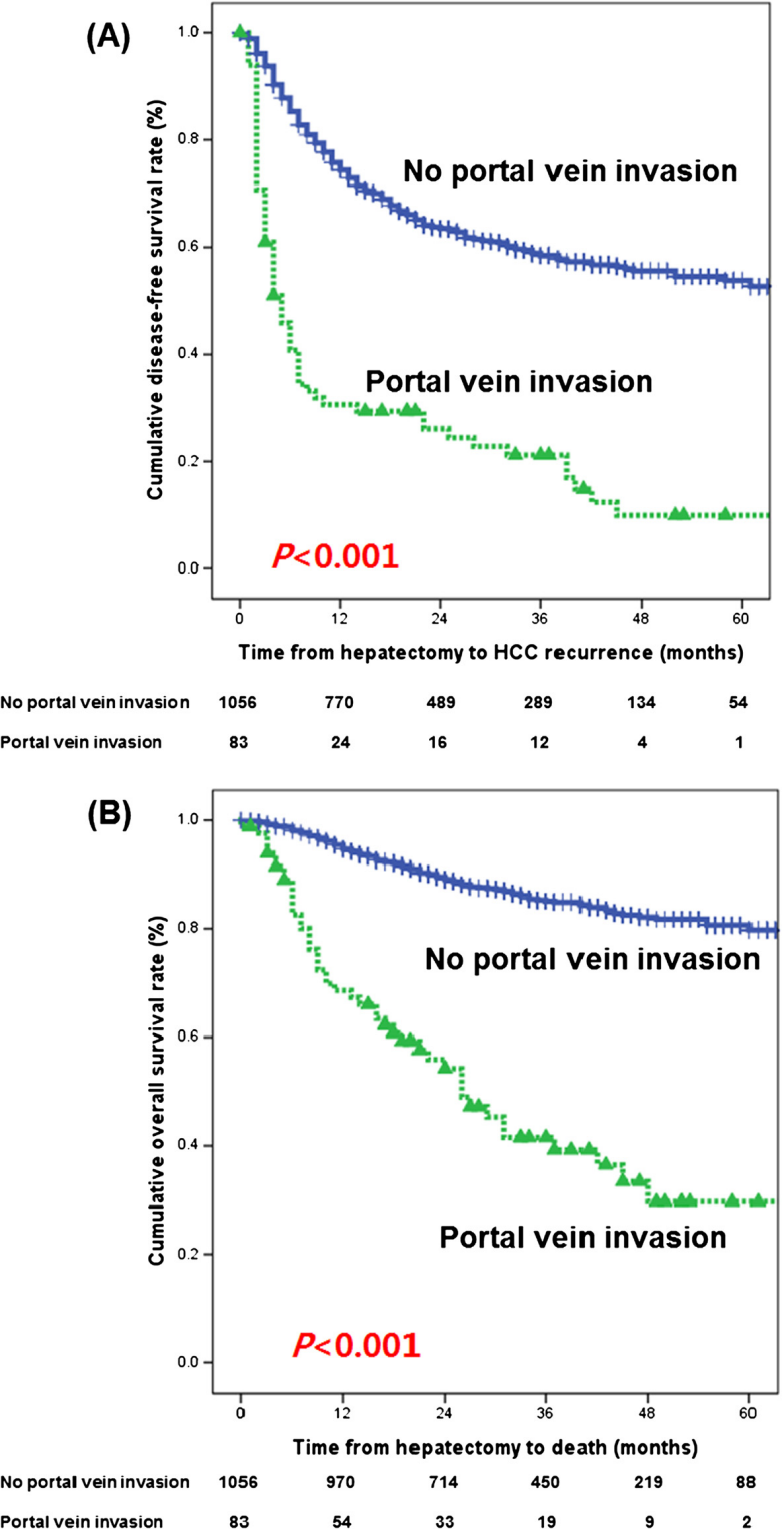
**(A) Disease-free survival rate and (B) overall survival rate in HCC patients according to portal vein invasion.** The disease-free survival curve and overall survival curve in HCC patients with malignant portal invasion were inferior to those in HCC patients without portal vein invasion.

For the HCC patients with malignant portal vein invasion who underwent liver resection, the median disease-free survival was 4.5 months and the overall survival was 25 months. The 1-year, 2-year, and 3-year disease-free survival rates and overall survival rates in HCC patients with malignant portal vein invasion were 30.6%, 26.1%, and 21.2%, and 68.6%, 54.2%, and 41.6%, respectively, but those in HCC patients without portal vein invasion were 74.4%, 63.5%, and 58.5%, and 95.5%, 89.6%, and 85.1%, respectively (*P* <0.001 and *P* <0.001, respectively) (Figure 1).

### Recurrence after surgical resection

The incidence of tumor recurrence was 80.7% (67/83) in HCC patients with malignant portal vein invasion and 38.8% (410/1056) in HCC without portal vein invasion. The initial detection site was lung (50.7%) in HCC patients with malignant portal vein invasion and liver (62.0%) in HCC patients without portal vein invasion. There was a difference in liver or lung as the initial detection site of tumor recurrence between the two groups (*P* <0.001 and *P* <0.001, respectively) (Table 3). However, no significant difference was found in the recurrence in bone, brain, and peritoneum between the two groups.

**Table 3:** Initial detection site for recurrence after surgical resection

	**Recurrence**		
**Sites**	**HCC with PV invasion (*n*=67)**	**HCC without PV invasion (*n*=410)**	***P*value**
Liver	18 (26.9%)	254 (62.0%)	<0.001
Lung	34 (50.7%)	95 (23.2%)	<0.001
Bone	7 (10.4%)	22 (5.4%)	0.161
Brain	0 (0%)	5 (1.2%)	0.364
Peritoneum	8 (11.9%)	34 (8.3%)	0.351

Radiofrequency ablation (RFA) in HCC patients without portal vein invasion was used more often because of high incidence of recurrence in liver, compared with HCC patients with malignant portal vein invasion (*P* <0.001). No HCC patients with portal vein invasion were treated with liver resection due to recurrence. Liver transplantation, TACE, sorafenib, radiation, and chemotherapy as treatments for tumor recurrence were similar in both groups (Table 4).

**Table 4:** Treatments for HCC recurrence

	**Recurrence**		
**Treatments on recurrence**	**HCC with PV invasion (*n*=67)**	**HCC without PV invasion (*n*=410)**	***P*value**
Liver resection	0 (0%)	18 (4.4%)	0.091
Liver transplantation	3 (4.5%)	7 (1.7%)	0.154
RFA	6 (9.0%)	125 (30.5%)	<0.001
TACE	39 (58.2%)	242 (59.0%)	0.894
Sorafenib	12 (17.9%)	67 (16.3%)	0.725
Radiation	9 (13.4%)	42 (10.2%)	0.400
Chemotherapy	5 (7.5%)	23 (5.6%)	0.573

RFA, Radiofrequency ablation; TACE, Transarterial chemoembolization.

### Prognostic factors for recurrence in HCC with malignant portal vein invasion after surgery

Univariate analysis showed that increased AFP level, decreased serum albumin level, serosa involvement, and intrahepatic metastasis were closely associated with tumor recurrence after liver resection (Table 5). On multivariate analysis of the prognostic factors for disease-free survival in HCC patients with malignant portal vein invasion, only the CRP level (hazard ratio, 1.133; 95% confidence interval, 1.028–1.249; *P*=0.012) was a significant independent predictor of tumor recurrence after liver resection.

**Table 5:** Risk factors for tumor recurrence in HCC patients with portal vein invasion by univariate analysis

**Variables**	**Hazard ratio**	**95% confidence interval**	***P*value**
Gender - Female	0.657	0.313–1.376	0.265
Age	0.973	0.946–1.000	0.052
AFP	1.000	1.000–1.000	0.005
PIVKA-II	1.001	1.000–1.001	0.128
Locoregional therapy	1.495	0.848–2.634	0.165
White blood cells	0.961	0.835–1.107	0.582
Hemoglobin	1.010	0.853–1.195	0.909
Platelet counts	1.000	0.998–1.003	0.821
INR	6.056	0.254–144.219	0.266
Albumin	0.533	0.290–0.980	0.043
Total bilirubin	1.047	0.654–1.676	0.849
AST	1.005	0.999–1.011	0.103
ALT	1.002	0.996–1.008	0.565
ALP	1.002	0.995–1.008	0.643
Creatinine	0.427	0.091–2.004	0.281
Estimated GFR	1.015	0.997–1.033	0.102
GGT	1.000	0.998–1.001	0.739
CRP	1.133	1.028–1.249	0.012
Operation (non-anatomical)	1.473	0.863–2.515	0.156
Hepatectomy (minor)	0.922	0.367–2.315	0.863
Maximum tumor size	1.003	0.997–1.010	0.282
Grade (3 and 4)	0.627	0.311–1.267	0.194
Cirrhosis	1.426	0.879–2.314	0.150
Capsule formation	1.020	0.353–2.941	0.971
Capsular invasion	0.715	0.439–1.165	0.178
Bile duct invasion	0.752	0.302–1.873	0.540
Serosa involvement	5.863	1.372–25.058	0.017
Intrahepatic metastasis	1.787	1.098–2.910	0.020
Multicentric occurrence	0.048	0.00–195.191	0.474
Margin (mm)	1.006	0.986–1.026	0.566

AFP, Alpha-fetoprotein; ALP, Alkaline phosphate; ALT, Alanine aminotransferase; AST, Aspartate aminotransferase; CRP, C-reactive protein; GFR, Estimated glomerular filtration rate; GGT, Gamma-glutamyl transferase; INR, International normalized ratio; PIVKA-II, Protein induced by vitamin K antagonist-II.

## Discussion

The prognosis for patients who have HCC with malignant portal vein invasion remained extremely poor in this study, even though a few long-term survivors were observed. Several studies have shown that tumor size, high grade, the presence of fibrous capsule, and platelet counts are strongly associated with portal vein invasion [8-10]. In the present study, increased ALP levels, increased maximum tumor size, and intrahepatic metastasis were independent predictors of malignant portal vein invasion in HCC by multivariate logistic regression analysis. Therefore, these factors may be helpful in predicting malignant portal vein invasion before surgery for HCC.

Many studies reported that an increase in the serum CRP level was associated with a shorter survival in patients with various malignancies, including multiple myeloma [11], ovarian cancer [12], colorectal [13], and esophageal cancer [14]. For the serum CRP levels associated with HCC, high CRP was linked to the diffuse type of HCC and elevated preoperative CRP levels in patients with HCC associated with tumor size and portal vein invasion, and predicted recurrence after curative resection and a poor surgical outcome [15,16].

The basis for the relationship between elevated CRP and poor prognosis is unclear and there are several possible explanations. An elevated CRP level may reflect a non-specific inflammatory response to tumor necrosis or local tissue damage, which may be indicative of a favorable environment for the establishment and growth of tumor. The serum level of vascular endothelial growth factor (VEGF), an angiogenic factor, is increased in the presence of raised CRP concentration [17]. Angiogenesis plays an important role in tumor growth and is associated with a poor outcome [18]. This creates a microenvironment that favors tumor angiogenesis, proliferation, growth, and metastases.

Tumor invasion in the remnant liver after surgery is common in HCC patients with malignant portal vein invasion [19]. However, our study revealed that lung metastasis was common in HCC patients with malignant portal vein invasion after surgery, while liver metastasis was common in HCC patients without portal vein invasion.

Although intrahepatic recurrence predominates, probably because of the early spread of neoplasm and metachronous multicentric carcinogenesis, several effective therapeutic modalities can control recurrent disease (such as repeated hepatectomy, TACE, percutaneous ethanol injection therapy, microwave coagulation therapy, and RFA) [16,20]. Compared with frequent intrahepatic recurrence, the incidence of distant metastasis is relatively low in HCC [21]. However, extrahepatic metastasis, such as lung, was higher than intrahepatic metastasis in HCC with portal vein invasion. The poor prognosis for patients with extrahepatic metastasis of HCC occurs because it is an indicator of an aggressive primary HCC [22].

Our study revealed that chest CT must be regularly performed in HCC patients with malignant portal vein invasion after surgical resection. In addition, patients who had preoperatively elevated CRP levels, should be closely monitored after hepatectomy because of a poor prognostic factor of tumor recurrence.

## Conclusion

In conclusion, our study reveals that increased ALP levels, increased maximum tumor size, and intrahepatic metastasis were independent predictors of malignant portal vein invasion in HCC. CRP levels are closely associated with the predisposing factor of tumor recurrence in HCC patients with malignant portal vein invasion after surgical resection, and lung metastasis is common.

## Competing interests

The authors declare no conflicts of interest.

## Authors’ contribution

JMK: data collection, analysis, and interpretation, manuscript writing; JWJ and SWP: data design and interpretation; CHDK, JSK: data collection and analysis; JBP, JHL, SJK, and CKP: data collection. All authors read and approved the final manuscript.

## References

[B1] Song IH Kim KS Current status of liver diseases in Korea: hepatocellular carcinoma Korean J Hepatol 2009 Suppl 6 S50 S59 20037280 10.3350/kjhep.2009.15.S6.S50

[B2] Llovet JM Burroughs A Bruix J Hepatocellular carcinoma Lancet 2003 362 1907 1917 10.1016/S0140-6736(03)14964-1 14667750

[B3] Matsumata T Kanematsu T Takenaka K Yoshida Y Nishizaki T Sugimachi K Patterns of intrahepatic recurrence after curative resection of hepatocellular carcinoma Hepatology 1989 9 457 460 10.1002/hep.1840090320 2537789

[B4] Jonas S Bechstein WO Steinmuller T Herrmann M Radke C Berg T Settmacher U Neuhaus P Vascular invasion and histopathologic grading determine outcome after liver transplantation for hepatocellular carcinoma in cirrhosis Hepatology 2001 33 1080 1086 10.1053/jhep.2001.23561 11343235

[B5] Villa E Moles A Ferretti I Buttafoco P Grottola A Del Buono M De Santis M Manenti F Natural history of inoperable hepatocellular carcinoma: estrogen receptors’ status in the tumor is the strongest prognostic factor for survival Hepatology 2000 32 233 238 10.1053/jhep.2000.9603 10915729

[B6] Forner A Reig ME de Lope CR Bruix J Current strategy for staging and treatment: the BCLC update and future prospects Semin Liver Dis 2010 30 61 74 10.1055/s-0030-1247133 20175034

[B7] Edmondson HA Steiner PE Primary carcinoma of the liver: a study of 100 cases among 48,900 necropsies Cancer 1954 7 462 503 10.1002/1097-0142(195405)7:3<462::AID-CNCR2820070308>3.0.CO;2-E 13160935

[B8] Miyata R Tanimoto A Wakabayashi G Shimazu M Nakatsuka S Mukai M Kitajima M Accuracy of preoperative prediction of microinvasion of portal vein in hepatocellular carcinoma using superparamagnetic iron oxide-enhanced magnetic resonance imaging and computed tomography during hepatic angiography J Gastroenterol 2006 41 987 995 10.1007/s00535-006-1890-2 17096068

[B9] Adachi E Maeda T Kajiyama K Kinukawa N Matsumata T Sugimachi K Tsuneyoshi M Factors correlated with portal venous invasion by hepatocellular carcinoma: univariate and multivariate analyses of 232 resected cases without preoperative treatments Cancer 1996 77 2022 2031 10.1002/(SICI)1097-0142(19960515)77:10<2022::AID-CNCR9>3.0.CO;2-S 8640665

[B10] Hagiwara S Kudo M Kawasaki T Nagashima M Minami Y Chung H Fukunaga T Kitano M Nakatani T Prognostic factors for portal venous invasion in patients with hepatocellular carcinoma J Gastroenterol 2006 41 1214 1219 17287901 10.1007/s00535-006-1950-7

[B11] Terpos E Szydlo R Apperley JF Hatjiharissi E Politou M Meletis J Viniou N Yataganas X Goldman JM Rahemtulla A Soluble receptor activator of nuclear factor kappaB ligand-osteoprotegerin ratio predicts survival in multiple myeloma: proposal for a novel prognostic index Blood 2003 102 1064 1069 10.1182/blood-2003-02-0380 12689925

[B12] Hefler LA Concin N Hofstetter G Marth C Mustea A Sehouli J Zeillinger R Leipold H Lass H Grimm C Tempfer CB Reinthaller A Serum C-reactive protein as independent prognostic variable in patients with ovarian cancer Clin Cancer Res 2008 14 710 714 10.1158/1078-0432.CCR-07-1044 18245530

[B13] Nozoe T Matsumata T Kitamura M Sugimachi K Significance of preoperative elevation of serum C-reactive protein as an indicator for prognosis in colorectal cancer Am J Surg 1998 176 335 338 10.1016/S0002-9610(98)00204-9 9817250

[B14] Shimada H Nabeya Y Okazumi S Matsubara H Shiratori T Aoki T Sugaya M Miyazawa Y Hayashi H Miyazaki S Ochiai T Elevation of preoperative serum C-reactive protein level is related to poor prognosis in esophageal squamous cell carcinoma J Surg Oncol 2003 83 248 252 10.1002/jso.10275 12884238

[B15] Lin ZY Wang LY Yu ML Chen SC Chuang WL Hsieh MY Tsai JF Chang WY Role of serum C-reactive protein as a marker of hepatocellular carcinoma in patients with cirrhosis J Gastroenterol Hepatol 2000 15 417 421 10.1046/j.1440-1746.2000.02149.x 10824887

[B16] Hashimoto K Ikeda Y Korenaga D Tanoue K Hamatake M Kawasaki K Yamaoka T Iwatani Y Akazawa K Takenaka K The impact of preoperative serum C-reactive protein on the prognosis of patients with hepatocellular carcinoma Cancer 2005 103 1856 1864 10.1002/cncr.20976 15779015

[B17] Xavier P Belo L Beires J Rebelo I Martinez-de-Oliveira J Lunet N Barros H Serum levels of VEGF and TNF-alpha and their association with C-reactive protein in patients with endometriosis Arch Gynecol Obstet 2006 273 227 231 10.1007/s00404-005-0080-4 16208475

[B18] Fondevila C Metges JP Fuster J Grau JJ Palacin A Castells A Volant A Pera M p53 and VEGF expression are independent predictors of tumour recurrence and survival following curative resection of gastric cancer Br J Cancer 2004 90 206 215 10.1038/sj.bjc.6601455 14710231 PMC2395306

[B19] Chen XP Qiu FZ Wu ZD Zhang ZW Huang ZY Chen YF Zhang BX He SQ Zhang WG Effects of location and extension of portal vein tumor thrombus on long-term outcomes of surgical treatment for hepatocellular carcinoma Ann Surg Oncol 2006 13 940 946 10.1245/ASO.2006.08.007 16788755

[B20] Minagawa M Makuuchi M Takayama T Kokudo N Selection criteria for repeat hepatectomy in patients with recurrent hepatocellular carcinoma Ann Surg 2003 238 703 710 10.1097/01.sla.0000094549.11754.e6 14578733 PMC1356149

[B21] Hong SS Kim TK Sung KB Kim PN Ha HK Kim AY Lee MG Extrahepatic spread of hepatocellular carcinoma: a pictorial review Eur Radiol 2003 13 874 882 12664129 10.1007/s00330-002-1519-7

[B22] Uchino K Tateishi R Shiina S Kanda M Masuzaki R Kondo Y Goto T Omata M Yoshida H Koike K Hepatocellular carcinoma with extrahepatic metastasis: clinical features and prognostic factors Cancer 2011 117 4475 4483 10.1002/cncr.25960 21437884

